# Metal halide perovskite nanomaterials: synthesis and applications

**DOI:** 10.1039/c6sc04474c

**Published:** 2016-12-16

**Authors:** Son-Tung Ha, Rui Su, Jun Xing, Qing Zhang, Qihua Xiong

**Affiliations:** a Division of Physics and Applied Physics , School of Physical and Mathematical Sciences , Nanyang Technological University , Singapore 637371 . Email: Qihua@ntu.edu.sg; b NOVITAS , Nanoelectronics Centre of Excellence , School of Electrical and Electronic Engineering , Nanyang Technological University , Singapore 639798; c Department of Materials Science and Engineering , College of Engineering , Peking University , Beijing 100871 , P. R. China

## Abstract

Nanomaterials refer to those with at least one dimension being at the nanoscale (*i.e.* <100 nm) such as quantum dots, nanowires, and nanoplatelets. These types of materials normally exhibit optical and electrical properties distinct from their bulk counterparts due to quantum confinement or strong anisotropy. In this perspective, we will focus on a particular material family: metal halide perovskites, which have received tremendous interest recently in photovoltaics and diverse photonic and optoelectronic applications. The different synthesis approaches and growth mechanisms will be discussed along with their novel characteristics and applications. Taking perovskite quantum dots as an example, the quantum confinement effect and high external quantum efficiency are among these novel properties and their excellent performance in applications, such as single photon emitters and LEDs, will be discussed. Understanding the mechanism behind the formation of these nanomaterial forms of perovskite will help researchers to come up with effective strategies to combat the emerging challenges of this family of materials, such as stability under ambient conditions and toxicity, towards next generation applications in photovoltaics and optoelectronics.

## Abstract 2

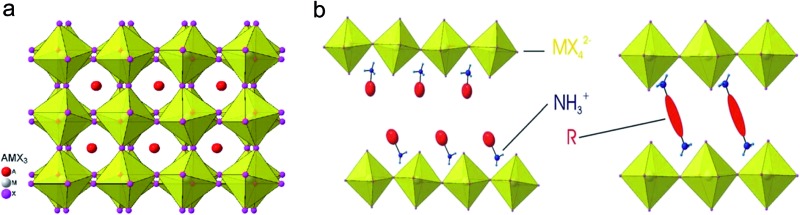
The different synthesis approaches and growth mechanisms of metal halide perovskites will be discussed along with their novel characteristics and applications.

## 1. Introduction

Nanomaterial research is one of the most important branches in modern physics where many quantum physics theories are experimented and validated. For instance, semiconductor quantum dots exhibit excellent exciton confinement and a near unity external quantum yield of photoluminescence. Another example is carbon nanotubes, which are an ideal quantum wire system for physicists to study electronic confinement in one dimension. In 2004, Andre Geim and Konstantin Novoselov, who were later awarded the Nobel Prize in Physics in 2010, experimentally isolated graphene – a single layer of hexagonal-structured carbon atoms.^1^ The discovery of this truly two dimensional (2D) material blasted a new wave in research at the global scale, leading the low dimensional materials to their “golden age”. Since then, many other families of 2D materials have been explored, such as boron nitride,^2,3^ transition metal dichalcogenide (TMD),^4–7^ and black phosphorus.^8,9^

So what are the properties that set aside nanomaterials from their bulk counterparts? First of all, when a material has one or more reduced dimensions, it can have a very high surface area to volume ratio. Consequently, the surface state of that material becomes more important and even dominant (*e.g.* in quantum dots). Moreover, the constraint of dimensionality in semiconductors leads to various quantum size effects, which can significantly change the energy spectrum of electrons and their behavior. In some systems, such as MoS_2_ and WS_2_, lowering the number of layers down to a monolayer will change the nature characteristics of the materials from indirect to direct bandgap. In graphene, the quantum mechanical description of the Bloch wave of electrons renders a new kind of quasiparticle, which moves like an electron that has completely lost its mass, following an analogous description of the Dirac equation. These new phenomena that only happen at low dimensionality are responsible for their extraordinary electronic, optical, thermal, mechanical, and chemical properties, which may be applied in a wide range of applications.

Recently, metal halide perovskites have received substantial attention from the research community due to their applications in solar cells,^10–12^ lasers,^13–15^ light emitting diodes (LED),^16–18^ water splitting,^19,20^ laser cooling^21^ *etc.* The material is now considered as the most promising material for the future of opto-electronics because of its high performance, low cost, and abundance.^22^ Lead halide perovskites were firstly used in the dye-sensitized solar cell configuration in 2009 by Miyasaka and colleagues with an initial efficiency of only 3.8%,^23^ and were later employed in the all-solid configuration in 2012 by Kim *et al.* ^24^ Since then, the performance of perovskite solar cells has been dramatically improved mostly by material and interface engineering, which boosted the efficiency over 20%.^25^ The perovskite material exhibits excellent optical and electrical properties, such as a high absorption coefficient, strong photoluminescence, low trap-state density, and long carrier diffusion length.^13,26–28^ Even though organic–inorganic hybrid perovskites have been synthesized and known for over 100 years, the first study of electrical properties for tin-based perovskite was reported by Mitzi *et al.* in 1994 as a new family of semiconductor materials.^29^ After that, the perovskites have been used in various forms of devices, such as field effect transistors (FETs), and LEDs with limited performance.^30–32^ The attention towards this material was only raised again when it was applied in a solar cell in 2009,^23^ which later turned out to be one of the most effective light absorber materials for solar cells to date.

**Fig. 1:**
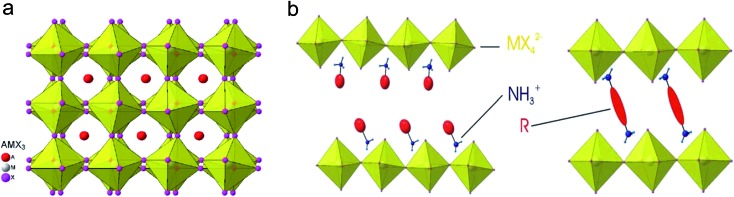
Structure of 3D and 2D perovskites:^33^ (a) structure of AMX_3_ 3D perovskite; (b) structure of layered perovskites with (left) monoammonium (RNH_3_^+^) or (right) diammonium (^+^H_3_N–R-NH_3_^+^) organic cations.

Metal halide perovskites are mainly classified into two categories based on their crystal structure motif. The first one is called a 3D-structured perovskite with the general chemical formula AMX_3_ and the other is called a 2D-structured or layered perovskite with the formula A_2_MX_4_, where M is a divalent metal (such as Pb or Sn), X is a halide (Cl, Br, or I), and A is a cation which can be inorganic (*e.g.* Cs^+^) or organic (*e.g.* CH_3_NH_3_^+^, C_4_H_9_NH_3_^+^, C_6_H_5_–C_2_H_4_NH_2_^+^, *etc.*). We should not be confused by the term 3D and 2D here, which are related to the structural motifs of the material while the other 0D–3D refer to the dimensions of materials. The term “perovskite” originated from the similarity in crystal structure of the material to the perovskite crystal CaTiO_3_. In this case, the divalent metal M is surrounded by six halogen atoms in an octahedral structure, and the cation A is either located in the center of an eight MX_6_ octahedral network (in the case of 3D perovskite) or sandwiched between corner-sharing MX_6_ octahedral layers (in 2D perovskite) as illustrated in Fig. 1.^33^ Due to its natural layered structure, where each layer of MX_6_ octahedra is only connected to each other by a weak van der Waals force, the 2D-structured perovskites are much easier to prepare in the 2D form, either by mechanical exfoliation or a chemical method. Indeed, Dou *et al.* has reported large scale synthesis of monolayer (C_4_H_9_NH_3_)_2_PbBr_4_ by a chemical method.^34^ Many other efforts have been made to prepare and characterize nanomaterial forms of the perovskites.^35–38^ In our group, we have prepared nanoplatelets and nanowires of perovskites by vapor phase synthesis to realize their excellent natural cavity in optical lasing.^14,39–41^ The high crystallinity and optical quality of the nanoplatelets also enabled the experimental demonstration of laser cooling in the perovskite material.^21^ The high external quantum efficiency of colloidal quantum dots of perovskites further boosted their performance in LEDs.^17,42^ Recently, the idea of hetero-structuring perovskite 2D sheets and other 2D materials, such as graphene or TMD, is becoming an interesting concept for integrating this material into low dimensional optoelectronics.

In this perspective, we aim at presenting recent breakthroughs in the preparation and characterization of metal halide perovskite nanomaterials (*i.e.* quantum dots, nanowires, and nanoplatelets). In the following sections, we would like to start from the synthesis of each type of perovskite nanomaterial, since this may be the first obstacle for any extensive material research to be made. We will review both chemical and physical methods and discuss their advantages as well as disadvantages. We will also discuss the growth mechanism to understand the formation of the material in its nanosized forms. Next, we will discuss their novel optical, electronic properties and their applications in various optoelectronic devices. Finally, we conclude the whole area and provide an outlook for the future development of these materials.

## 2. Synthesis and growth mechanism of metal halide perovskite nanomaterials

### 2.1 Perovskite quantum dots

Organic–inorganic perovskite colloidal nanoparticles (NPs) were firstly synthesized by Schmidt *et al.* ^43^ The NPs were prepared by a simple and reliable method under an ambient atmosphere, in which a DMF solution of CH_3_NH_3_Br and PbBr_2_ reacted and precipitated in an acetone solution in the presence of long chain alkyl ammonium bromide, oleic acid (OLA) and octadecene (ODE). They proposed the growth mechanism as while the methylammonium cations are embedded in the voids of the corner-sharing PbX_6_ octahedra, the long alkyl chain cations only fit the periphery of the octahedra with their chains dangling outside it. Thus, these ammonium ions would act as the capping ligands to limit the growth of the colloidal NPs extending in three dimensions. The XRD patterns showed a cubic phase of the MAPbBr_3_ NPs and the HRTEM images showed the spherical morphology of the NPs with an average size of 6 nm. The MAPbBr_3_ colloidal NPs exhibited an absorption peak at 525 nm and a PL peak at 527 nm which is blueshifted compared to the absorption peak at 550 nm and PL peak at 570 nm of the MAPbBr_3_ bulk crystal. The colloidal NPs showed a high PL quantum yield (PLQY) of 20% in toluene and remain stable in a series of aprotic, moderate polarity, organic solvents for more than three months. Next, they further optimized the synthesis of MAPbBr_3_ colloidal NPs by tuning the ratio of MABr, PbBr, octylammonium bromide (OABr) and ODE.^44^ Finally, the best colloidal NPs with a particle size of 5.5 nm and PLQY of 83% were obtained by using a larger ratio of the ammonium and PbBr_2_, but in the absence of OLA.

**Fig. 2:**
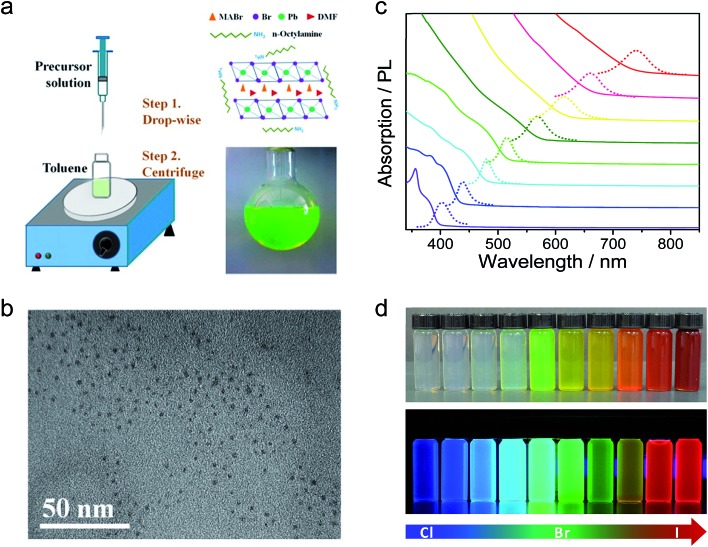
(a) Schematic illustration of the reaction system and process for the LARP technique and typical optical image of the colloidal MAPbBr_3_ solution. (b) HRTEM image of MAPbBr_3_ colloidal NPs. (c) Optical absorption and PL spectra of perovskites with different halide components. (d) Digital image of the perovskite colloidal solutions in toluene under ambient light and an UV lamp, light emission from 438 to 660 nm. Reprinted with permission from ref. 17 and 45, copyright 2015, 2016 American Chemical Society.

A similar precipitation method was also used to synthesize mixed halide perovskite MAPbX_3_ colloidal NPs with different Br/I and Br/Cl compositions (Fig. 2).^45^ The PL spectra of these mixed halide perovskites can be finely tuned from 407 to 734 nm by varying the content of halogen cations and their absolute PLQYs can be reach 50–70%. Typically, MAPbBr_3_ NPs have an average diameter of 3.3 nm and a PLQY of 70%. The exciton binding energy of MAPbBr_3_ NPs was calculated to be 375 meV, which is much larger than that of the bulk counterpart of 65 meV. Rogach *et al.* reported a synthesis of MAPbBr_3_ NPs with the variation of the temperature of the bad solvent between 0–60 °C.^46^ The MAPbBr_3_ NP size can be controlled by the temperature; 1.8 nm, 2.8 nm, and 3.6 nm average diameters were obtained under 0, 30, and 60 °C, respectively. The PL spectra were tuned from 470 nm (0 °C) to 520 nm (60 °C) and the PLQYs of the series colloidal NPs started at 74% for the 0 °C sample and steadily increased, reaching as high as 93% for the 60 °C sample. However, these phenomena are still not enough to confirm the quantum confinement of the perovskite colloidal NPs. That is because the long chain alkyl ammonium was used as ligands to synthesize the perovskite NPs, which might form quasi-2D perovskites with blueshifted PL spectra.^47,48^ Our group also reported the synthesis of the perovskite MAPbX_3_ colloidal NPs with emission from 403 to 740 nm.^17^ However, we used different precursor solutions; DMF and γ-butyrolactone as the solvent, octylamine as ligands and without OLA. Here, the absence of the OLA can keep the colloidal solution more stable, even enabling storage for several months. It can be deduced from the XRD patterns and HRTEM images that the as-synthesized perovskite colloidal NPs exhibit a typical amorphous phase, which is attributed to free PbX_6_ (X = Br or DMF anion) dispersed in the DMF and γ-butyrolactone mixed solution. When the precursor solution was injected into toluene, PbX_6_ units would aggregate and reprecipitate, but the crystallization process would be blocked by the octylamine. Notable, the HRTEM was conducted at a low acceleration voltage of 60 kV to exclude the possible recrystallization of the amorphous perovskite under a high acceleration voltage. Although the reprecipitation method is a fast and facile strategy to synthesize highly luminescent MAPbX_3_ colloidal NPs, the yield of the product is very limited due to the formation of aggregated clusters together with the NPs.

**Fig. 3:**
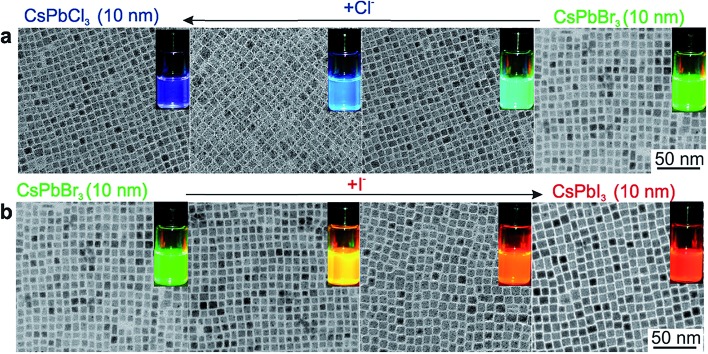
Transmission electron microscopy (TEM) images of ∼10 nm CsPbX_3_ NCs after treatment with various quantities of (a) chloride and (b) iodide anions. The insets show the evolution of emission colors under an UV lamp. Reprinted with permission from ref. 50, copyright 2015, American Chemical Society.

The high PLQY of perovskite MAPbX_3_ colloidal NPs motivated the preparation of inorganic CsPbX_3_ (X = Cl, Br, I, and mixed Cl/Br and Br/I) NPs. Protesescu *et al.* synthesized CsPbX_3_ colloidal NPs for the first time by injecting the precursor Cs-oleate into a PbX_2_ solution containing OLA, OA, trioctylphosphine (TOPO), and ODE at high temperature as in the case of CdS/Se quantum dots.^49^ The CsPbX_3_ colloidal NPs exhibited a cubic shape (4–15 nm edge lengths) and cubic perovskite crystal structure. Through compositional modulations and quantum size-effects, the emission spectra are also tunable over the entire visible spectral region of 410–700 nm. The PL of the CsPbX_3_ NCs shows narrow emission line widths of 12–42 nm (from blue to red) and high PLQYs of 50–90%. Within the effective mass approximation, they estimated the effective Bohr diameters of Wannier–Mott excitons and the binding energies for CsPbCl_3_ (5 nm, 75 meV), CsPbBr_3_ (7 nm, 40 meV), and CsPbI_3_ (12 nm, 20 meV). Nedelcu *et al.* and Akkerman *et al.* further observed fast anion exchange in CsPbX_3_ (X = Cl, Br, I) perovskite NPs at low temperature (Fig. 3).^50^ By adjusting the halide ratios in the colloidal nanocrystal solution, the bright PL could be tuned over 410–700 nm while maintaining high quantum yields of 20–80% and narrow emission line widths of 10–40 nm. Furthermore, the fast inter-nanocrystal anion-exchange was also demonstrated between the CsPbCl_3_, CsPbBr_3_, and CsPbI_3_ NPs, leading to uniform CsPb(Cl/Br)_3_ or CsPb(Br/I)_3_ compositions. After that, there are many reports on CsPbX_3_ colloidal NPs synthesized based on this methods, but with different crystal phases. For example, Swarnkar *et al.* synthesized CsPbBr_3_ colloidal NPs following the above methods, but obtained orthorhombic phase CsPbBr_3_.^51^ It should be noted that the colloidal NPs had broader XRD peaks and the cubic and orthorhombic phases exhibited very closed XRD patterns, so it is difficult to distinguish these phases.

Inspired by the methodology for synthesis of perovskite MAPbX_3_ colloidal NPs, Li *et al.* extended the supersaturated recrystallization method to inorganic perovskite CsPbX_3_.^52^ Although crystallized at RT, these colloidal NPs have superior optical properties to those formed at high temperature, including PLQYs of 80%, 95%, and 70% for red, green, and blue PLs, and a 90% retention rate after aging for 30 days under ambient conditions. Deng *et al.* used a reprecipitation strategy to systematically manipulate the shape of CsPbX_3_ colloidal NPs, such as spherical colloidal NPs and nanocubes, by using different ligands.^53^ De Roo *et al.* studied the dynamic ligand of the inorganic perovskite CsPbX_3_.^54^ They found that compared to classical chalcogenide quantum dots, CsPbX_3_ is more ionic in nature and the interactions with capping ligands are also more ionic and labile. Therefore, ligand binding to the NP surface is highly dynamic and easily lost during the isolation and purification procedures. However, when a small amount of both oleic acid and oleylamine is added, the NPs can be purified, maintaining optical, colloidal, and material integrity. A high amine content in the ligand shell would increase the quantum yield due to the improved binding of the carboxylic acid. Most recently, Swarnkar *et al.* ^55^ reported the synthesis of ambient-stable cubic-phase quantum dots of CsPbI_3_, a phase that was previously known to be stable only at high temperature. They have developed an improved synthetic route and purification approach that prevent the CsPbI_3_ quantum dots from transforming their as-synthesized cubic phase to orthorhombic. The difference in their method compared to the conventional method is that they used methyl acetate (*i.e.* antisolvent) to remove excess unreacted precursor without causing agglomeration, resulting in stable cubic CsPbI_3_ quantum dots. The material has been used to fabricate solar cells and LEDs, which exhibited the highest power conversion efficiency of 10.77% for all-inorganic perovskite solar cells to date.^55^

### 2.2 Perovskite nanowires (1D)

As lead halide perovskites have triggered a revolution in photovoltaic solar cells, much effort has been directed to the synthesis of large area lead halide perovskite films due to their excellent optoelectronic properties.^11,16,56–61^ Nonetheless, lead halide perovskite nanowires are also of great importance due to their novel optical and electronic properties, as well as their potential as building blocks for various applications in optoelectronic devices.^15,39,62–65^ Over the past few decades, a lot of effort has been spent on the synthesis of II–VI and III–V semiconductor nanowires, which is mainly based on a chemical vapor deposition (CVD) process.^66–70^ This method is the most widely used approach due to its versatility and simplicity in many semiconductor systems. The CVD process is used to obtain nanowires primarily based on a vapor–liquid–solid (VLS) mechanism, utilizing noble metal films as the catalyst to drive the one dimensional crystal growth. However, this VLS method is rarely adopted in the growth of perovskite nanowires, which could be possibly attributed to the low growth temperature of perovskites.

**Fig. 4:**
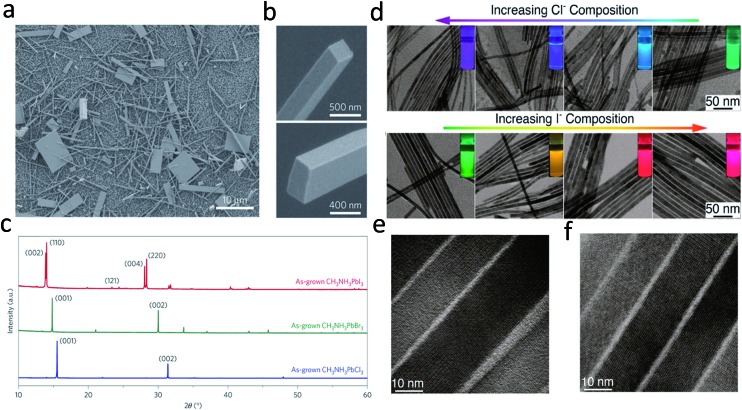
(a) and (b) SEM images of CH_3_NH_3_PbI_3_ nanostructures. (c) PXRD patterns of as-grown CH_3_NH_3_PbX_3_ (X = I, Br, Cl) NWs. (d) TEM images of CsPbX_3_ NWs with various degrees of conversion with chloride and iodide anions. The insets show the evolution of emission color (UV excitation, *λ* = 365 nm) upon forming mixed-halide CsPb(Br/Cl)_3_ and CsPb(Br/I)_3_ NWs. HRTEM images of (e) Cl- and (f) I-exchange NWs. Reprinted with permission from ref. 15 and 74, copyright 2015, Nature Publishing Group and 2016, American Chemical Society.

Compared with previous III–VI and II–VI semiconductors based on a VLS mechanism, the facile solution processed synthesis of lead halide perovskites could be the most attracting point for low cost applications.^13,27,71–73^ Much effort has been devoted to synthesizing lead halide perovskite nanowires by solution methods.^15,62,65,74^ Large area high quality single crystal methylammonium (MA) lead halide perovskite nanowires were reported in the pioneering work on nanowire lasers by Zhu and his co-workers.^15^ In this work, no surfactant ligand is used for the controlled anisotropic crystal growth. A lead acetate (PbAc_2_) solid thin film was firstly deposited on a glass substrate by using a PbAc_2_·3H_2_O aqueous solution. This PbAc_2_ solid film was then immersed in a CH_3_NH_3_X (X = I, Br, Cl or mix halide precursors) solution in isopropanol in an ambient environment for over 20 h. Consequently, high quality methylammonium lead halide perovskite nanowires were obtained with lengths up to 20 μm and flat rectangular end facets (Fig. 4b). The width of the rectangular end facets is typically a few hundred nanometers. The corresponding X-ray power diffraction pattern (Fig. 4c) confirms the pure tetragonal phase of single-crystalline CH_3_NH_3_PbI_3_ nanowire, the cubic phase of single-crystalline CH_3_NH_3_PbBr_3_ and single-crystalline CH_3_NH_3_PbCl_3_ nanowires. A dissolution–recrystallization growth mechanism was proposed in the above growth procedure. A CH_3_NH_3_PbX_3_ thin film immediately forms at the surface, which severely suppresses the diffusion of CH_3_NH_3_^+^ ions. This results in a large amount of PbAc_2_ remaining on the substrate and slowly dissolving into the solution until PbX_4_^2–^ reaches the equilibrium point for precipitation with CH_3_NH_3_^+^ and further reaches supersaturation to crystallize into CH_3_NH_3_PbX_3_. Consequently, a low precursor concentration (and thus supersaturation) was maintained for the anisotropic one-dimensional crystal growth of CH_3_NH_3_PbX_3_.^15,75^

Following this work, a solution processed method with surfactant ligand was reported to control the synthesis of all-inorganic perovskite CsPbX_3_ (X = Cl, Br, I or mixture of halides) nanowires.^65^ This method was widely used for previous metal and inorganic semiconductor nanowires, using a surfactant ligand as the capping ligand to initiate one dimensional crystal growth. The CsPbX_3_ nanowires were synthesized under an air-free environment by reacting cesium oleate with lead halide in the presence of oleic acid and oleylamine in octadecene at 150–250 °C for 5–10 min. Oleylamine here serves as a capping ligand of Pb^2+^, reducing the activity of the Pb^2+^ precursor. Meanwhile, oleylamine preferentially binds to certain facets of CsPbX_3_, leading to the anisotropic, one dimensional crystal growth to obtain perovskite nanowires. The as-synthesized CsPbX_3_ perovskite nanowires exhibit diameters less than 12 nm and lengths up to 5 μm. However, the production yield of the nanowires is not high and a lot of byproducts, such as nanoplatelets and nanocrystals, are produced along with the nanowires. By replacing the original oleic acid with octylamine, the nanowire yield has been dramatically improved, yielding up to 90% after simple purification.^74^ Moreover, by an anion exchange reaction, CsPbCl_3_ and CsPbI_3_ nanowires can be transformed from CsPbBr_3_ nanowires by reacting with other halide precursors, as shown in Fig. 4d and e. The nanowires transformed from CsPbBr_3_ nanowires exhibit a high quantum efficiency up to 83% and 30% for CsPbI_3_ and CsPbCl_3_, respectively. As shown in Fig. 4e and f, single crystalline features of CsPbI_3_ and CsPbCl_3_ are still maintained after transformations, confirmed by their HRTEM images.^74^ High crystallinity and excellent optical properties enable perovskite nanowires to serve as promising candidates for optoelectronic applications.

**Fig. 5:**
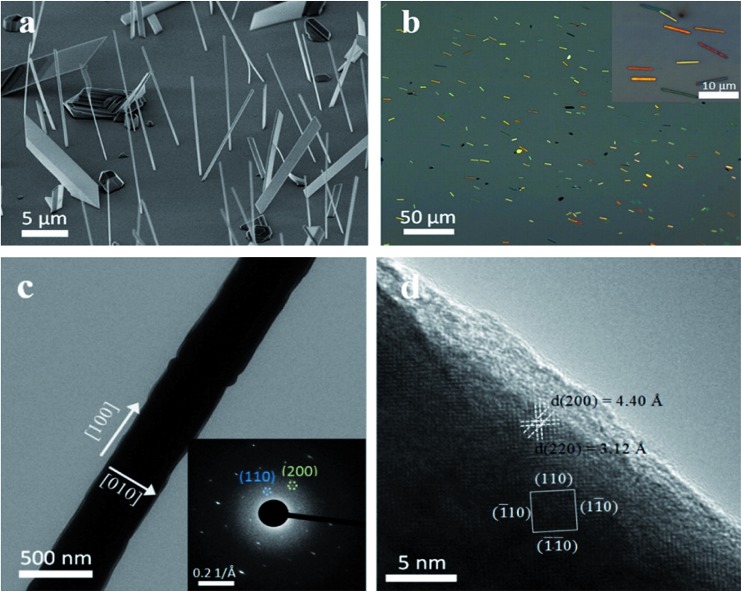
(a) SEM image of PbI_2_ nanowires grown on the silicon substrate. (b) Optical microscopy image of CH_3_NH_3_PbI_3_ nanowires on the silicon substrate. Inset in (b) is the magnified image. (c) TEM and (d) HRTEM images of CH_3_NH_3_PbI_3_ nanowire. Inset in (c) is its corresponding SAED pattern. Reprinted with permission from ref. 39, copyright 2015, American Chemical Society.

In addition to solution phase synthesis of perovskite nanowires, high quality perovskite nanowires have also been obtained through a vapor phase synthesis. Xing *et al.* utilized a two-step vapor phase method to synthesize high crystalline quality methylammonium lead iodide perovskite nanowires.^39^ Briefly, PbI_2_ nanowires were firstly synthesized on a silicon dioxide substrate by placing the PbI_2_ precursor in a chemical vapor deposition furnace. Fig. 5a shows the scanning electron microscopy image of PbI_2_ nanowires. PbI_2_ nanowires were vertically grown on the silicon dioxide substrate and possess rectangular or near square cross sections. Those nanowires were subsequently transferred to another substrate by pressing the new substrate on top of the original substrate with vertically grown nanowires, as shown in Fig. 5b. Consequently, nanowires on the new substrate were placed in the furnace for conversion into perovskites by intercalating CH_3_NH_3_I into the interval sites of PbI_6_ octahedra layers. The nanowire morphology was still maintained after the conversion. The converted perovskite nanowires exhibited single crystalline properties and grew along the [100] direction, which were confirmed by their SAED patterns (Fig. 5c and d). However, for this kind of two-step method, the reaction needs to be carefully controlled as an incomplete reaction will result in polycrystalline products or the dual-existence of perovskite and PbI_2_. With the morphology acting as an active resonator, these vapor phase grown perovskite nanowires exhibited an excellent performance in optically pumped lasing.

### 2.3 Perovskite nanoplatelets

**Fig. 6:**
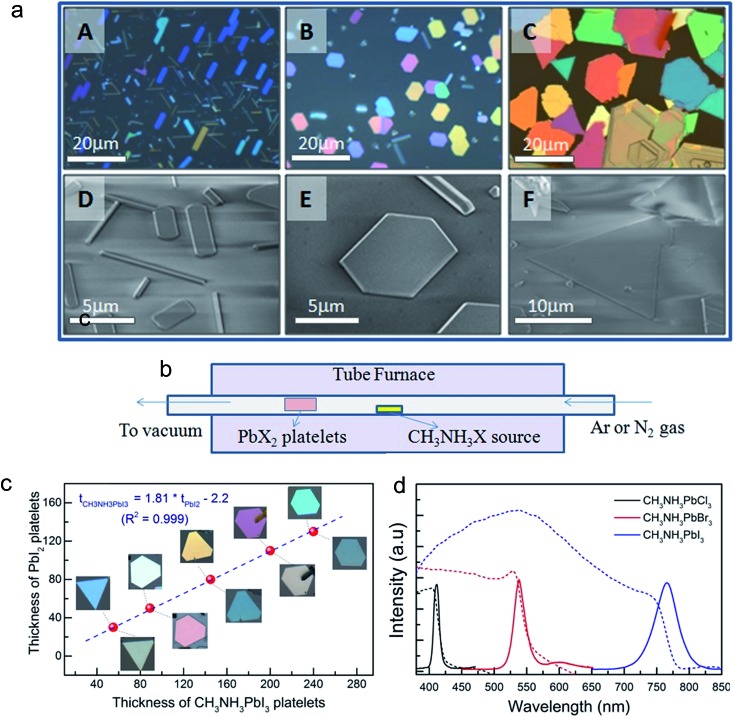
Morphological characterizations of lead halide nano-platelets as-grown on a muscovite mica substrate: (a) optical (above) and SEM (below) images of lead halides: A, D: PbCl_2_; B, E: PbBr_2_; C, F: PbI_2_. (b) Schematic of the synthesis setup using a home-built vapor-transport system. (c) Thickness of PbI_2_ platelets before (images above data line) and after being converted to CH_3_NH_3_PbI_3_ (images below data line). Note that the color of the PbI_2_ platelets changes, corresponding to the change in thickness (as measured by AFM). (d) Optical properties of different lead halide perovskites (CH_3_NH_3_PbX_3_). Reprinted with permission from ref. 41, copyright 2014, Advance Optical Materials.

Among all low dimensional forms, 2D materials are probably the most important one for practical applications in optoelectronic devices. Because its lateral size is at macro-scale, a 2D material can be easily integrated with other existing electronic materials in a scalable way. The stacking of different 2D materials in a designable configuration can create sophisticated devices, such as FETs, photodetectors, LEDs, and solar cells, and is usually difficult to achieve in a single crystal of 0D or 1D materials. Nanoplatelets, a quasi-form of 2D material, have a lateral size of tens of micrometers and a thickness of a few to a few tens of nanometers. In research, it is usually easier to prepare 2D materials in the form of nanoplatelets or nanoflakes rather than a millimeter scale film. The fact that the nanoplatelet is defect-free, a high quality single crystal, and has a large lateral size that is enough for any optical or electronic characterization makes it a preferred subjected for 2D material studies. The perovskite material is no exception. Our group was among the first to report the synthesis of lead halide perovskite nanoplatelets using a home-built vapor deposition system as illustrated in Fig. 6.^41^ In this two-step method, first lead halide crystals were grown on a muscovite mica substrate utilizing the van der Waals epitaxial growth mechanism (Fig. 6a). The super smooth surface of the freshly cleaved mica and low lattice mismatch between the lead halide lattice constants and those of the mica endure the horizontal growth of the crystals, which results in nanoplatelet formation. The as-grown lead halide nanoplatelets were then intercalated with methylammonium halide using the same vapor deposition system, which converted the nanoplatelets into respective perovskites (Fig. 6b). After conversion, the perovskite nanoplatelet thickness was increased by the factor of ∼1.8 while retaining lateral dimensions comparable to their PbI_2_ counterparts (Fig. 6c), which is in good agreement with the theoretical calculation based on the crystal structures of the two compounds. The resulting perovskite nanoplatelets were shown to have excellent optical properties and high crystallinity. The CH_3_NH_3_PbI_3_ nanoplatelet was shown to have twice the electron diffusion length of the solution processed film.

Liu *et al.* ^76^ introduced a different approach using a hot casting aqueous solution of PbI_2_ on a SiO_2_ substrate to form PbI_2_ nanoplatelets and then converted them to perovskite by intercalating methylammonium halide using a vapor deposition system. Due to the layered structure of PbI_2_, the crystals tend to self-assemble into platelets in its aqueous solution during the hot casting process. Thanks to their layered structure, the PbI_2_ nanoplatelets can also be prepared by mechanical exfoliation from the bulk crystals as reported by Cheng *et al.* ^77^ The similarity between the three methods above is that they are all two-step methods in which the physical growth of lead halide nanoplatelets is followed by vapor phase intercalation of methylammonium halide. These approaches, especially the first one – using a vapor deposition system for all steps, have the advantage of using high purity sources of precursors and eliminating the possible contamination and by-product production during synthesis. However, due to the two-step growth and the solid phase intercalation of lead halide, the crystallinity of the resulting perovskite nanoplatelets may not be as good as in the single crystal growth in the solution phase.

**Fig. 7:**
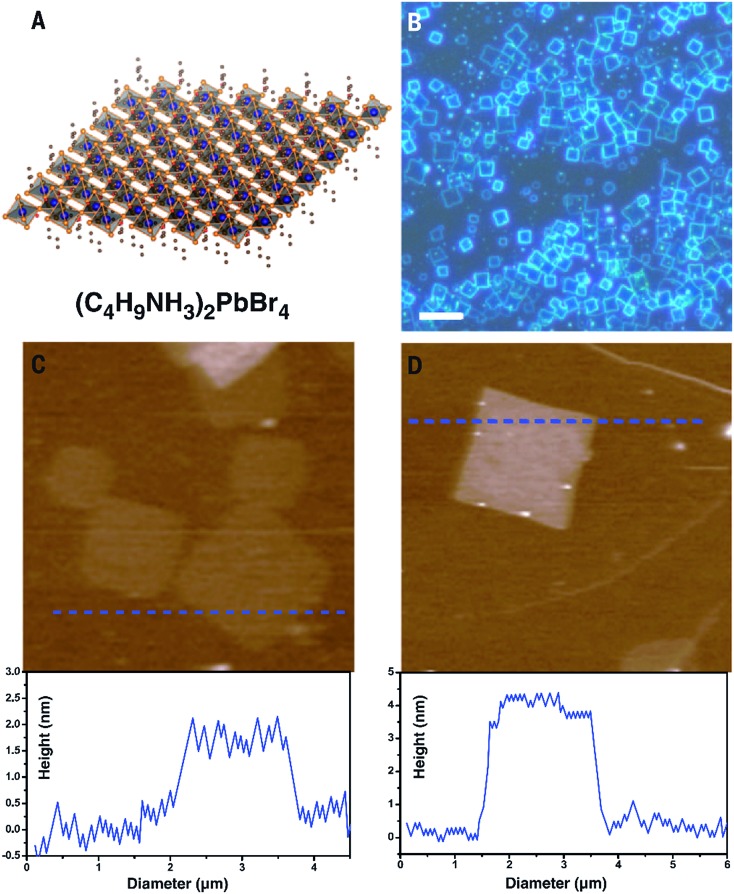
Synthesis of atomically thin 2D (C_4_H_9_NH_3_)_2_PbBr_4_ crystals. (A) Structural illustration of a single layer (C_4_H_9_NH_3_)_2_PbBr_4_ (blue balls, lead atoms; large orange balls, bromine atoms; red balls, nitrogen atoms; small orange balls, carbon atoms; H atoms were removed for clarity). (B) Optical image of the 2D square sheets. Scale bar, 10 mm. (C) AFM image and height profile of several single layers. The thickness is around 1.6 nm (*T* 0.2 nm). (D) AFM image and height profile of a double layer. The thickness is around 3.4 nm (*T* 0.2 nm). Reprinted from ref. 34, Copyright 2015, Science Publishing Group.

Wang *et al.* reported a simple method to grow single crystal perovskite microplates.^78^ Perovskite in *N*,*N*-dimethylformamide (DMF) solution was drop-casted onto a hydrophobic substrate (*i.e.* ITO scribed with polyethylene film). The formation of self-assembled microplates was controlled by the volume ratio of perovskite solution and an anti-solvent – dichloromethane (DCM). The dispersion of DCM vapor in the perovskite solution induced the nucleation and subsequent growth of perovskite microplates. The above methods were applied to 3D-structured perovskites where the thickness of the nanoplatelets was in the order of tens or even hundreds of nanometers. The nature of the 3D crystalline structure of these perovskites makes them difficult to prepare at mono- or even few layers thick. The other type of perovskite, which has a 2D layered structure, may be a better candidate to study the 2D form. Indeed, Dou *et al.* ^34^ has reported a solution method to prepare monolayer square sheets of (C_4_H_9_NH_3_)_2_PbI_4_ which opened up a new direction to study perovskite at an atomically thin level comparable with other 2D materials such as graphene or TMD. The result is summarized in Fig. 7.

In this method, a very diluted solution of perovskite in a co-solvent system of chlorobenzene (CB), acetonitrile and DMF was dropped onto a Si/SiO_2_ substrate prior to mild heating of the substrate to induce crystal growth of the perovskite square sheets. The co-solvent system played an important role as CB and acetonitrile help to reduce the solubility of perovskite in DMF and promote the crystallization. The diluted solution of perovskite (*i.e.* 10^–3^ M) was also critical to obtain monolayer sheets.

**Fig. 8:**
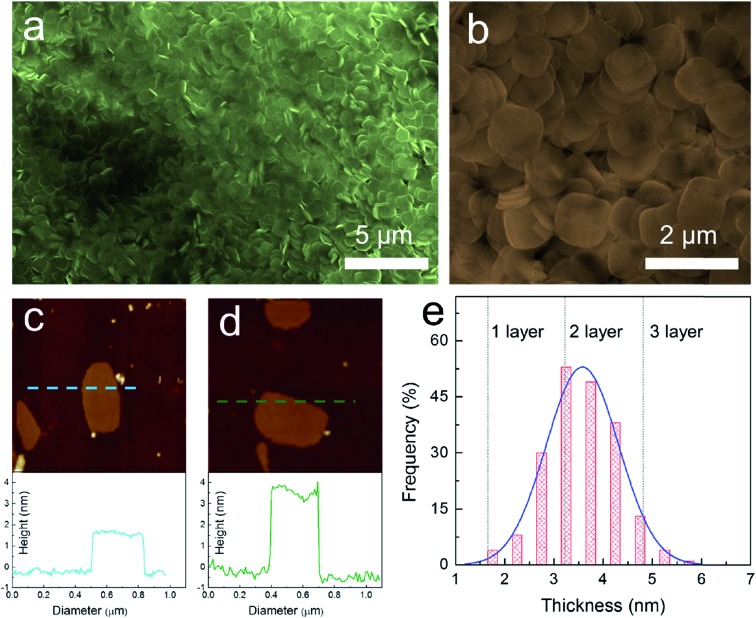
Synthesis of 2D ultrathin CsPbBr_3_ nanosheets: (a) low-magnification and (b) high-magnification SEM images. AFM image and height profile of (c) monolayer and (d) bilayer CsPbBr_3_ nanosheets with a thickness of 1.6 and 3.3 ± 0.2 nm, respectively. (e) Thickness distribution histograms for CsPbBr_3_ nanosheets prepared through solution-phase synthesis. Reprinted from ref. 79, copyright 2016, Advanced Materials.

One of the common methods to prepare nanosheets in the literature is to use a surfactant as a morphology assisting agent during the growth of a material in solution. Despite the fact that using a surfactant will result in contamination of the surface of the grown materials and may eventually reduce their performance in electronic properties, this is one of the few methods that provide high throughput and reproducibility. Song *et al.* ^79^ and Sun *et al.* ^53^ both applied similar approaches to synthesize nanoplatelets of cesium lead bromide perovskite (CsPbBr_3_). Both methods used long-chain alkyl amines such as oleylamine, dodecylamine, octylamine, and long-chain carboxylic acids such as oleic acid as co-surfactants. Instead of using cesium halide as a precursor like the other method, they used carboxylates of cesium such as cesium oleate or cesium stearate for better solubility in the hydrocarbon solvent, which was the medium for the reaction. The result is that they can synthesize nanoplatelets on a large scale with high monodispersity and control the thickness of the perovskite down to a monolayer, as shown in Fig. 8.^79^

## 3. Optical properties and applications of metal halide perovskite nanomaterials

### 3.1 Optical properties

#### Tunable emission

**Fig. 9:**
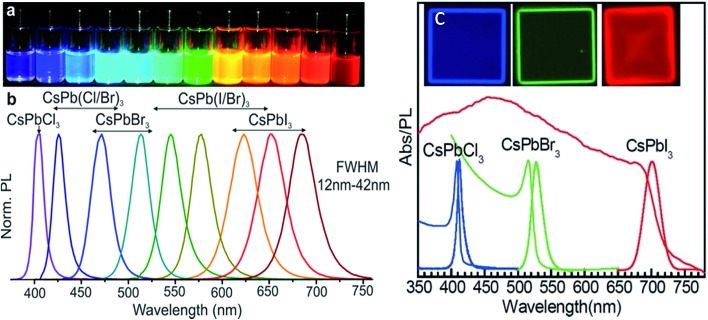
(a) Colloidal perovskite CsPbX_3_ NCs (X = Cl, Br, I) solutions in toluene under an UV lamp (*λ* = 365 nm); (b) representative PL spectra (*λ*_exc_ = 400 nm for all but 350 nm for CsPbCl_3_ nanocrystals). (c) Optical absorption and PL spectra of CsPbCl_3_, CsPbBr_3_, and CsPbI_3_ nanoplatelets. Inset: PL image of CsPbCl_3_, CsPbBr_3_, and CsPbI_3_ nanoplatelets. Reprinted with permission from ref. 40 and 49, copyright 2015, American Chemical Society and 2016, Advanced Material.

As perovskites have triggered a revolution in the solar cell research field, they have attracted a lot of attention in the past few years thanks to their excellent exciton and carrier properties. Apart from their superior performance in the solar cell research area, perovskites themselves also serve as outstanding light emitters in LEDs and laser applications.^14,18,42,80–86^ Compared with conventional III–V and II–VI semiconductors, one of the most attractive features of perovskites is their facile tunable emission throughout the whole visible range, achieved through controlling the stoichiometry. The emission of perovskite can be tuned by substituting halide elements, *e.g.*, from chloride to iodide. By substituting either the mixture of chlorides and bromides, or bromides and iodides, the emission of all inorganic-perovskite CsPbX_3_ (X = Cl, Br, I, or their mixture), including quantum dots and nanoplatelets, can be well tuned from 400 nm (blue) to 700 nm (red), which covers the whole visible region (as shown in Fig. 9).^40,49,87^ Another way to tune the emission is to replace the lead with other kinds of metal ions, or insert other kinds of organic molecules. The emission of perovskite can be further tuned to the near infrared or ultraviolet region.^81,88^

#### High quantum efficiency

Quantum efficiency, defined as the ratio of the number of converted photons to absorbed photons, is one of the most crucial properties for light emitters. High quantum efficiency usually signifies that most of the absorbed photons were converted through radiative recombination processes rather than non-radiative recombination processes. Perovskites are regarded as excellent light emitters due to their large absorption coefficient and high quantum efficiency.^50^ High quantum efficiencies up to 90% have been reported in both all-inorganic CsPbX_3_ and organic–inorganic methylammonium lead halide perovskite nanocrystals without any further surface treatment.^49,87^ In contrast, for conventional III–V and II–VI semiconductors, their nanocrystals usually suffer from surface defect states or donor–acceptor levels which strikingly reduce the quantum efficiency. The high quantum efficiency in perovskite is the result of a clear bandgap with negligible charge-trapping states, which greatly promote the exciton radiative recombination efficiency.^89^ With their high quantum efficiency, perovskites are promising alternatives for light emitting applications.

#### Quantum confinement effect

**Fig. 10:**
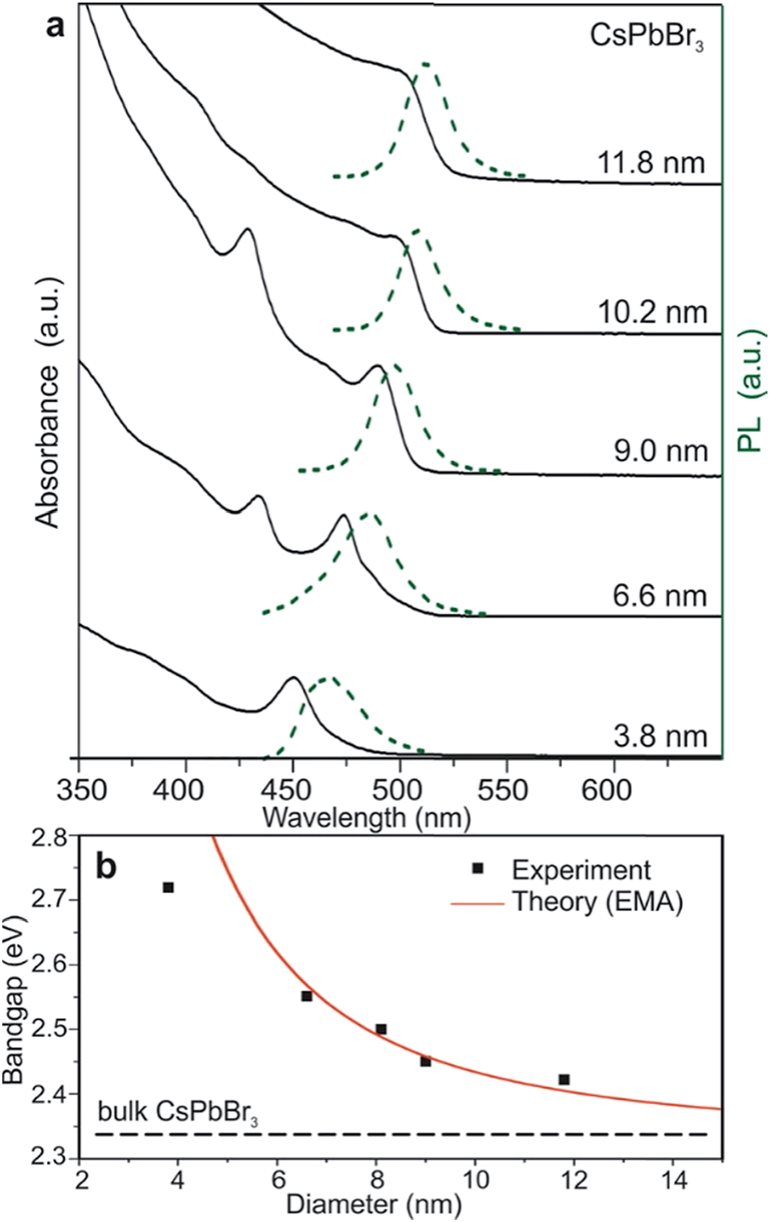
(a) Quantum-size effects in the absorption and emission spectra of 5–12 nm CsPbBr_3_ NCs. (b) Experimental *versus* theoretical (effective mass approximation, EMA) size dependence of the band gap energy. Reprinted with permission from ref. 49.

When the size of a semiconductor is too small to be comparable to the Bohr radius of excitons, quantum confinement could be observed in the optical properties of the semiconductor. Excitons are confined in all three spatial dimensions, which results in a transition from continuous to discrete energy levels. Consequently, the optical absorption and emission properties could be tuned by changing the size of the semiconductor. The quantum confinement effect is usually associated with nanocrystals and results in the blue shift of the bandgap with the decrease of the crystal size. In all-inorganic CsPbBr_3_ perovskite nanocrystals, the exciton Bohr radius is calculated to be 7 nm; the quantum confinement effect is quite prominent in CsPbBr_3_ perovskite nanocrystals when its size becomes comparable with the exciton Bohr radius. The emission of CsPbBr_3_ perovskite nanocrystals can be truly tuned from around 2.7 eV to 2.4 eV with the size changing from 4 nm to 12 nm which is in good agreement with the theoretical calculation, as shown in Fig. 10a and b.^49^ Similarly, the quantum confinement effect is also observed in organic–inorganic methylammonium lead halide perovskite nanocrystals.^87^ The quantum confinement effect provides a way to tune the emission of semiconductors, which results in potential for various light emitting applications.

### 3.2 Applications in optoelectronics

#### Optical lasing in perovskite

**Fig. 11:**
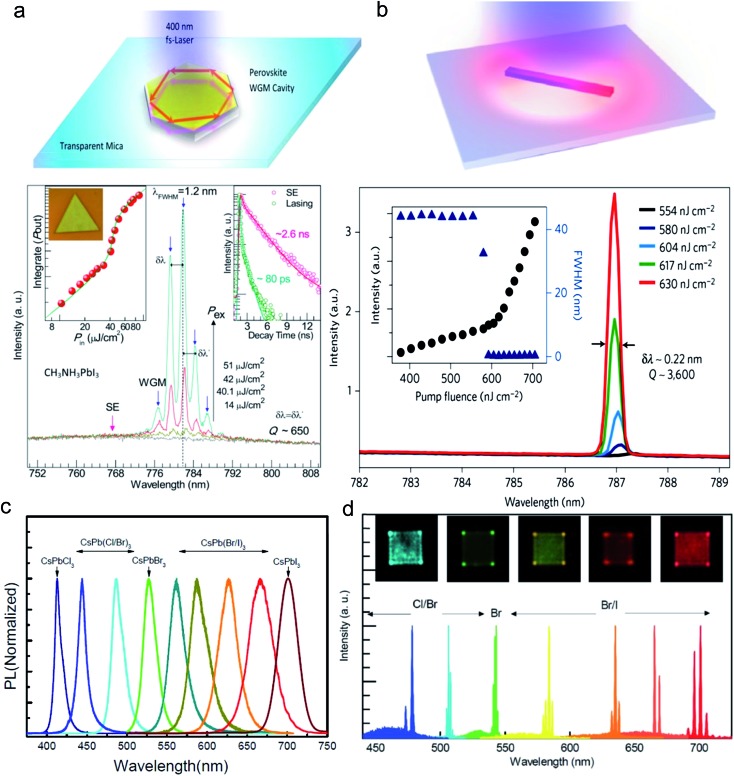
Optical lasing in lead halide perovskite nanoplatelets and nanowires. (a) Whisper-gallery mode lasing in a perovskite nanoplatelet cavity. (b) Fabry-Pérot lasing in a perovskite nanowire cavity. (c) Photoluminescence spectra of CsPbX_3_ (X = Cl, Br, I) nanoplatelets. (d) Wavelength tunability of perovskite lasing by changing the content of halide in CsPbX_3_ perovskite. Reprinted with permission from ref. 14, 15 and 40.

Metal halide perovskite is well-known for its high absorption coefficient and strong photoluminescence. When a high gain material is put in a suitable optical cavity, one can expect that lasing happens. Since the discovery of amplified spontaneous emission (ASE) in perovskite, many research studies have been done to explore their potential application in lasers. One of the early attempts was to put the material on top of a distributed Bragg reflector (DBR) which amplified the emission to achieve lasing. The other approach is to use a perovskite crystal as a naturally formed cavity. Our group was among the first to demonstrate that perovskite nanoplatelets can act as a whispering-gallery mode cavity and achieved lasing without the help of an artificial optical cavity (Fig. 11a).^14^ We also demonstrated that high crystalline perovskite nanowires can act as a Fabry-Pérot cavity to achieve lasing in the material.^39^ This is of interest for their potential applications in nanoscale optoelectronics. By manipulating the halide content in the perovskite composition, it is possible to precisely control the emission wavelength and achieve lasing over the entire visible spectrum (Fig. 11c and d).^40^ Many other researchers reported similar observations in nanoplatelets and nanowires with a high quality factor and low lasing threshold.

Zhu *et al.* demonstrated lasing in CH_3_NH_3_PbX_3_ with an exceptionally low threshold of only 220 nJ cm^–2^ (Fig. 11b), corresponding to a carrier density as low as 1.5 × 10^16^ cm^–3^. Another remarkable thing about the nanowires is that they have a very low number of carrier trapping sites and the estimated lasing quantum yield is close to 100%.^15^ We believe that in addition to high optical gain, the high quality crystalline structure of these perovskite nanowires and nanoplatelets is responsible for their high performance as a self-lasing material. With their exceptional coherent light emission and their ambipolar charge transport properties, the perovskite materials may someday be applied in electrically driven lasing.

#### Light emitting diodes

**Fig. 12:**
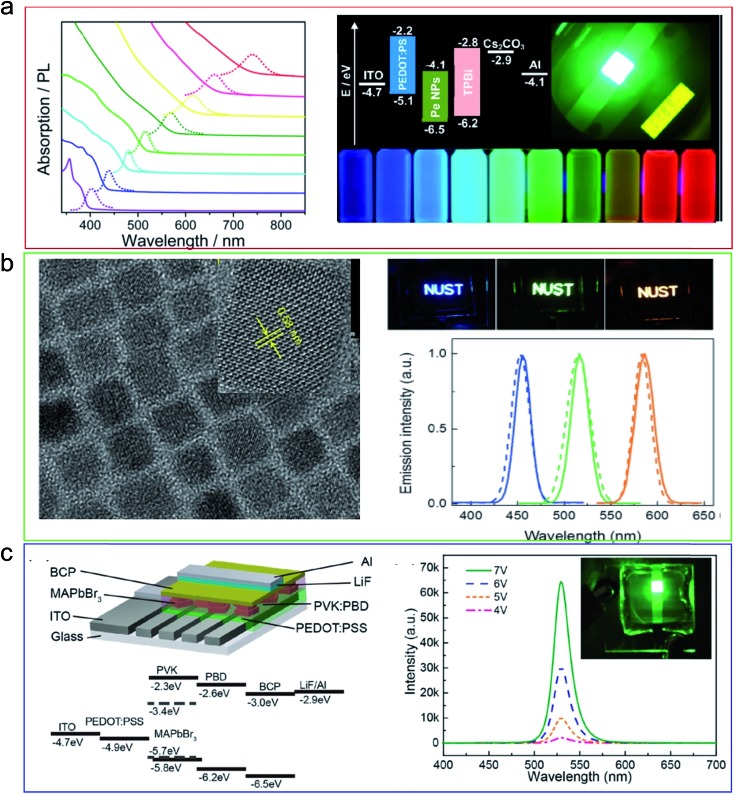
Low dimensional perovskite in LED applications. (a) LED from CH_3_NH_3_PbX_3_ quantum dots. (b) LED from CsPbX_3_ quantum dots. (c) LED from CH_3_NH_3_PbX_3_ nanoplatelets. Reprinted with permission from ref. 17, 90 and 91.

Beside their wide use in solar cell applications, metal halide perovskite is emerging as one of the most promising materials for light emitting diodes due to it being easy-to-prepare, having a low cost, and having a high performance. One important advantage of perovskites in LED applications is that they usually have high color purity with the full width half maximum of ∼15–25 nm for the electroluminescence spectra. Another advantage is the color tunability over the whole visible spectrum by simply changing the content of different halides within the compounds. For LED applications, a smaller grain size is preferable because it limits the exciton diffusion which in turn increases the possibility of radiative recombination. Thus, quantum dots seem to be a good candidate due to their strong luminescence and high external quantum yield. Indeed, many researchers have reported the use of perovskite quantum dot as active materials for LEDs and achieved exceptional performance when compared to the solution-prepared film. Our group also prepared amorphous quantum dots of perovskite with some novel properties and used them to fabricate LEDs with performances being among the highest of devices to date (Fig. 12a).^17^ All inorganic perovskites (*i.e.* CsPbX_3_) were also synthesized in the form of quantum dots for LED applications (Fig. 12b)^90^ to overcome the stability issues of organic–inorganic hybrid perovskite. Even though their performance in LEDs is lower than that of quantum dots, perovskite nanoplatelets have also been used to fabricate LEDs (Fig. 12c).^91^ The octylamine capped perovskite nanoplatelets showed an improvement in device stability and even allowed it to be fabricated in air. All of these achievements make perovskite a new generation of LED material for lighting and display applications.

#### Other applications

Low dimensional perovskites are being applied in many other optoelectronic applications such as FETs, photodetectors, and single photon emitters. Liu *et al.* ^76^ used perovskite nanoplatelets to fabricate a FET on a Si/SiO_2_ substrate. The *I*–*V* curve recorded for that device showed a linear dependence on the applied bias suggesting ohmic contact between perovskite nanoplatelets and the electrodes. The ratio of photocurrent and dark current can reach up to two orders of magnitude which the authors attributed to the strong light–material interaction as well as broad-band light harvesting capacity of the perovskite. Deng *et al.* ^35^ fabricated photodetectors based on a horizontal array of CH_3_NH_3_PbI_3_ nanowires on a glass substrate. The electrodes were patterned by a simple shadow mask with the active channel of 350 μm. The resulting photodetectors have a response time of 0.3 ms, a responsivity of 1.3 A W^–1^, and a detectivity of 2.5 × 10^12^ Jones, which are superior to those of the bulk perovskite and other inorganic nanowire photodetectors. Another interesting application of perovskite nanomaterials is the use of quantum dots as a single photon emitter at room temperature, as reported by Park *et al.* ^37^ The authors used CsPbX_3_ perovskite as a precursor material to synthesize quantum dots having cubic shapes and an average size of ∼10 nm. The perovskite quantum dots showed a strong photon anti-bunching of the emitted light and strong photoluminescence (PL) intensity fluctuation correlating with the PL lifetime. They attributed this phenomenon as “A-type blinking” which is commonly observed in a quantum dot system.

## 4. Conclusions and outlook

Metal halide perovskites represent a vast family of interesting semiconducting materials with exceptional optical and electronic properties. Unlike traditional III–V or II–VI semiconductors, there are thousands of combinations of perovskite compositions that can be easily synthesized. This provides a facile method to tune the characteristics of the material, such as the band-gap, conductivity, mobility, and so forth, which are very important for device configuration and optimization. The abundance of the material, low cost, and high performance of perovskite make this a promising material for future optoelectronic applications.

However, one of the biggest problems for this type of material is its stability. Due to the vulnerability of perovskite to surrounding environments such as moisture and oxygen, the performance of the devices may dramatically drop within only tens to hundreds of exposed hours, which casts a shadow towards their practical applications. Perovskite nanomaterials with high surface areas may seriously suffer from the effects of the environment. Thus, the need to develop a method to stabilize the material is inevitable either by chemical approaches such as capping agents, stabilizers or by physical approaches such as device encapsulation. It is well-known that 2D structured perovskites are normally more stable than their 3D counterparts. This is because the long chain organic cations in the 2D perovskite act like a protecting layer insulating MX_6_ octahedral structures from environmental agents (*i.e.* moisture, oxygen). However, these organic layers also hinder the charge transport in 2D perovskite resulting in their poor performance in optoelectronic applications compared to the 3D counterparts. If the conductivity of 2D structured perovskite can be improved, one can expect a high performance, stable perovskite device. Indeed, in a recent report in *Nature*, Tsai *et al.* demonstrated the use of a 2D–3D hybrid structural perovskite in a high performance and long-time stable solar cell device.^12^ By changing the composition of CH_3_NH_3_^+^ (3D component) and C_4_H_9_NH_3_^+^ (2D component) in the lead iodide perovskite formula, they were able to tune the crystal planes parallel to the substrate surface from 001 (in 2D perovskite) to 101 (in 2D–3D hybrid perovskite). Thus, the PbI_6_ octahedra network will come into direct contact with both the anode and cathode of the cell to facilitate efficient charge transport. The resulting cell exhibits an efficiency of 12.52% with no hysteresis and retained over 90% of its original efficiency after 3000 hours. This is a milestone in perovskite research, which is an ultimate solution to the biggest problem of the material. We can expect to see many efforts to synthesize perovskite nanomaterials with this hybrid formula and their applications in optoelectronics in the future.

There is still room for chemists to improve the optical and electronic properties of perovskite by tailoring the chemistry and crystallographic sciences. The next generation of this material may be truly multifunctional organic hybrid perovskites with application-driven compound design. We need to understand thoroughly the functions of each component in the perovskite formula such as organic cations, metal halide octahedra network and the effect of each on the entire crystal structure in order to formulate a better compound for different applications. This is an unusual but fantastic class of semiconductors where every characteristic (*i.e.* band structure, conductivity, mobility *etc.*) can be finely tuned to suit any requirement of a material. The future of the material is very much dependent on chemical scientists further exploring the thousands of unknown combinations of the perovskite out there.
